# Running an Internet Hospital in China: Perspective Based on a Case Study

**DOI:** 10.2196/18307

**Published:** 2021-09-16

**Authors:** Lihua Zhi, Pei Yin, Jingjing Ren, Guoqing Wei, Jun Zhou, Jun Wu, Qun Shen

**Affiliations:** 1 Department of Internet Hospital Office The First Affiliated Hospital Zhejiang University School of Medicine Hangzhou China; 2 Department of General Practice The First Affiliated Hospital Zhejiang University School of Medicine Hangzhou China; 3 Department of Medical Administration The First Affiliated Hospital Zhejiang University School of Medicine Hangzhou China

**Keywords:** internet hospitals, telemedicine, medical service, medical procedures, operation management, network security

## Abstract

Internet hospitals, as a new forum for doctors to conduct diagnosis and treatment activities based on the internet, are emerging in China and have become integral to the development of the medical field in conjunction with increasing reforms and policies in China’s medical and health system. Here, we take the Internet Hospital of the First Affiliated Hospital, Zhejiang University (FAHZU Internet Hospital) as an example to discuss the operations and functional positioning of developing internet hospital medical services in relation to physical hospitals. This viewpoint considers the platform operation, management, and network security of FAHZU Internet Hospital, and summarizes the advantages and limitations in the operation to provide a reference for other areas with interest in developing internet hospitals.

## Introduction

Internet hospitals are emerging in China as a new way for doctors to perform health care services and education by using telecommunications technology. Internet hospitals are similar to telemedicine, meaning that doctors who are registered in internet hospitals can conduct diagnosis and treatment activities on the internet, and provide subsequent visiting services such as online consultation, prescribing, and dispensing for patients diagnosed with common or chronic diseases in offline hospitals. Family physician services are also provided [1-3]. Internet hospitals are virtual hospitals that are initiated by the government, hospitals, or companies. Internet hospitals initiated by hospitals are equipped with a complete medical team of doctors, nurses, pharmacists, and managers, and patients can also receive examinations at offline hospitals. The scope of medical insurance is the same as that of offline hospitals, and the medical experience is very similar to that of physical hospitals [4].

The Chinese government issued the “Opinion on Actively Pushing Forward the Development of ‘Internet+’ Action” in July 2015, which clearly encouraged the development of internet hospitals as part of the “Health China” strategy [5]. In 2018, the “Opinion on Promoting the Development of ‘Internet+’ Healthcare,” “Administration Regulations on Internet Diagnoses and Treatments (Trial),” “Administration Regulations on Internet Hospitals (Trial),” and “Administration Specifications for Telemedicine Services (Trial)” were successively issued to propose specific requirements for operating and supervising the diagnosis and treatment activities performed by internet hospitals [6]. The government, hospitals, and enterprises in China are enthusiastic about building internet hospitals. The first internet hospital was founded at the end of 2014. As of May 2019, 158 internet hospitals were subordinate to various medical institutions in China [7,8].

Telemedicine models of care, in which doctors working in hospitals provide medical care over the internet using approved telehealth platforms, are used by many countries, but they differ with respect to the details of their operation. A selection of representative telehealth platform service providers from different countries is provided in Table 1 for comparison [9-12]. During the COVID-19 pandemic, many countries in addition to China have expanded the scope of diagnosis and treatment, including newly diagnosed patients, and expanded the medical insurance reimbursement limit. However, it is not clear whether this will become a long-term policy [9,13,14].

**Table 1:** Comparison of operation modes of main telemedicine operators in different countries.

Country	Platform	Operator	Insurance coverage (compared with offline)	Additional royalty	Involved diseases^a^
China	FAHZU^b^ Internet Hospital	Hospital	Consistent	Partly^c^	Follow-up after diagnosis of common or chronic diseases
United Kingdom	NHS^d^ App	Public body	Consistent	No	Determined by the GP^e^ practice
United States	American Well	Company	Inconsistent	Yes	Acute and chronic conditions, consultations regarding prevention and wellness services
Japan	CLINICS	Company	Consistent	Yes	Follow-up after diagnosis of chronic diseases

^a^During the COVID-19 epidemic, all countries except China expanded the scope of diagnosis and treatment, including the initial diagnosis of patients.

^b^First Affiliated Hospital, Zhejiang University.

^c^There is an extra charge for the expert clinic and image-text consultation clinic.

^d^NHS: National Health Service.

^e^GP: general practitioner.

As one of the top hospitals in China, the First Affiliated Hospital of Zhejiang University School of Medicine founded the first large-scale internet hospital in China in February 2016 (FAHZU Internet Hospital), and is representative of this type of hospital in China [15,16]. After 4 years of operation, more than 75,000 patients have received health care services via the internet hospital, leading to a wealth of experience in operating and managing this internet hospital [17,18]. Therefore, this paper aims to describe how an internet hospital in China is run from the perspectives of the service standards, treatment process, charges, platform operation, and management of FAHZU Internet Hospital.

### Background

FAHZU Internet Hospital is coordinated by the Internet Hospital Office, which is subordinate to the medical department of the offline hospital with hospital leaders as the primary individuals in charge of the project. The normal operation of the internet hospital is jointly supported by the Information Center, Nursing Department, Quality Management Department, Finance Department, Pharmaceutical Department, Promotion Center, and various inspection departments. In addition, corresponding support in internet technology, logistics, and distribution is offered through cooperation with third-party companies specializing in the internet and medicine. An internet hospital monitoring platform was constructed by the Zhejiang Province Health and Family Planning Commission at the beginning of 2019 to perform online/offline integrated monitoring of internet hospitals, and to ensure the safety and quality of the remote medical service.

FAHZU Internet Hospital provides subsequent visiting services for some patients with common and chronic diseases who receive a diagnosis in offline hospitals. The hospital can also guide patients who have severe illnesses that are not suitable for online consultation to the offline hospital for inspection, diagnosis, and treatment. Both patients and doctors can complete online inquiry diagnoses through their mobile phones or computers. Insurance also offers some types of coverage for internet hospitals.

### Health Care Services

**Figure 1:**
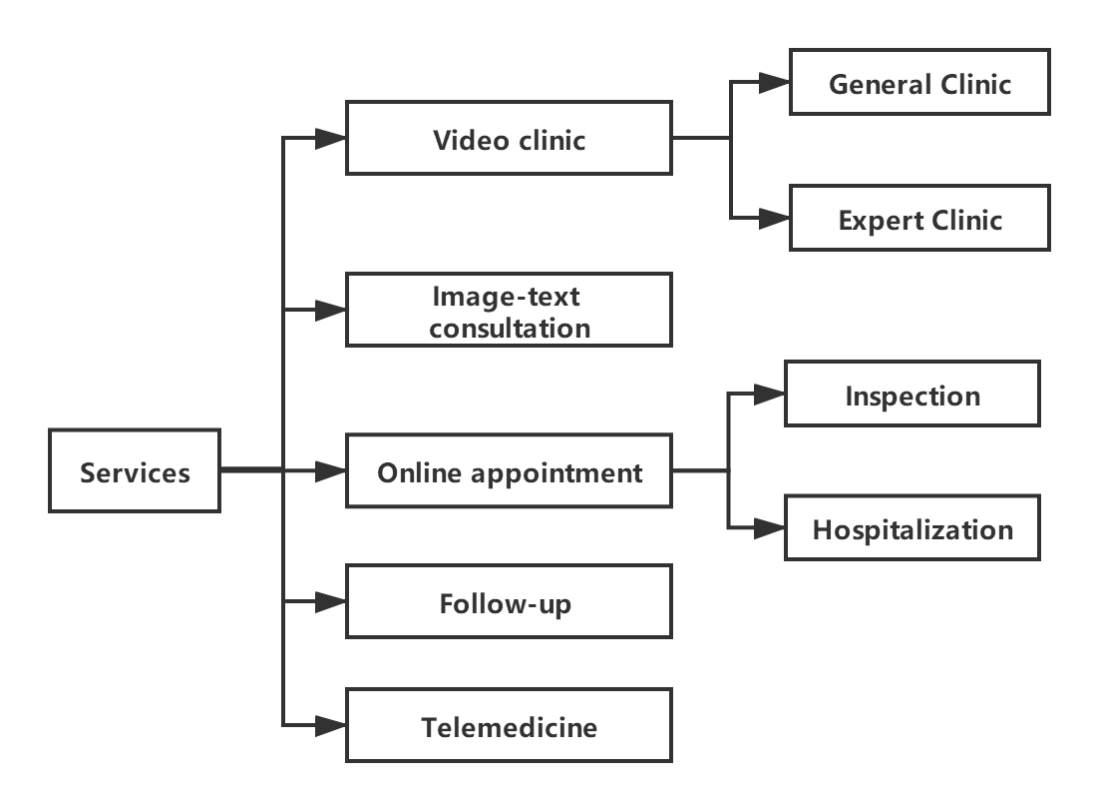
Various medical services provided in the internet hospital.

Various health services are provided in the internet hospital (Figure 1). Doctors provide patients with online services that include general clinics and expert clinics through face-to-face communication or image-text consultation with patients via remote video systems. With the same clinic hours as the offline hospital, doctors are uniformly assigned to be available at the web-based clinic by the responsible department. Patients register in real time and queue for service. The departments are dominated by common chronic diseases and consultation specialties (Table 2). The expert clinic and image-text consultation clinic are voluntarily administered by senior doctors who have worked independently for more than 3 years and are not limited by their specialties. The training is completed by the Internet Hospital Office. Doctors use their time outside work to provide medical services, and patients can choose the doctor they prefer. It is worth noting that no prescriptions are issued in the image-text consultation clinic since no defined diagnosis is made given the diagnosis limitation, and the charges for the services of the expert clinic and the image-text consultation clinic are priced independently by doctors. Although the clinic operates outside of routine working hours, which should be paid at the patient’s expense, the treatment charges are still covered by insurance.

**Table 2:** General clinic departments associated with the internet hospital.

Departments	Scope of services
Common chronic diseases departments^a^	Provide follow-up management services and health guidance, adjust and prescribe drugs, prescribe examinations, provide services for patients with common and chronic diseases
Health management	Provides health management guidance for healthy people and interpretation of routine physical examination reports
Pharmacy	Provides an online medication guide for patients
Nursing specialties consultation	Provides nursing consultation such as wound and stoma nursing for discharged patients and people with mobility difficulties, as well as nursing guidance for pregnant women, mothers, and babies
Fever clinic^b^	Screens out suspected COVID-19 patients and guides them to the hospital for diagnosis and treatment; patients who are judged to be infected with ordinary viruses are instructed on care at home

^a^Including the Departments of General Practice, Hepatitis Center, Gastroenterology, Cardiology, Endocrinology and Metabolism, Respiratory Medicine, Kidney Disease Center, Neurology, Rheumatology, Pediatrics, Mental Health, and Geriatrics.

^b^Only set up during the COVID-19 pandemic [19].

**Figure 2:**
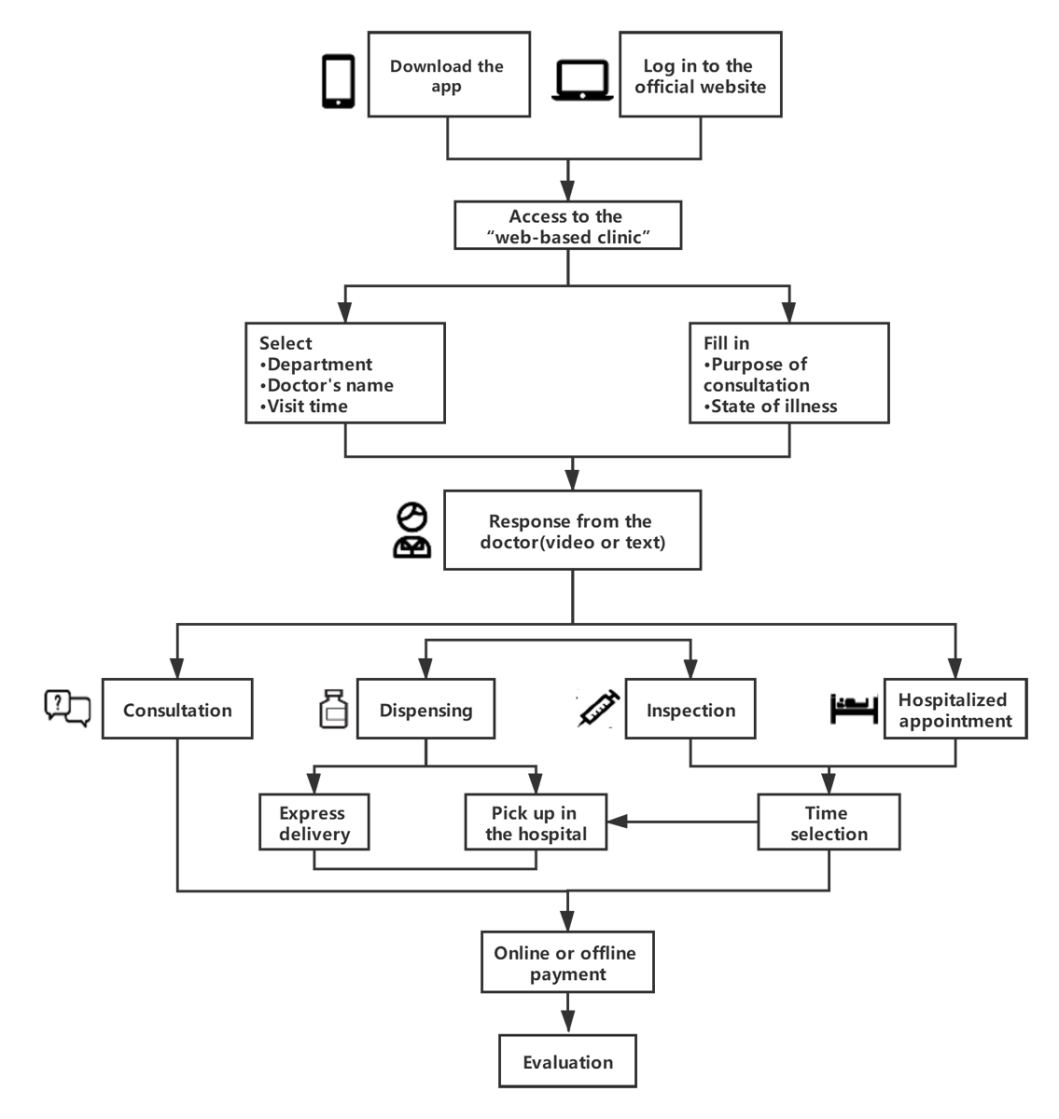
Flowchart of web-based clinic medical services for patients.

When a patient uses internet hospital services, they first download the app “Online FAHZU” via a mobile phone or log in to the official website of the First Affiliated Hospital, Zhejiang University via a PC to access the web-based clinic. The patient then selects the consultation department, expert, time, and purpose of consultation; fills in the medical record; and uploads relevant information. After payment via Alipay, patients wait for the doctor’s video invitation. After the video diagnosis is complete, patients can also check the diagnosis record and evaluate the service. If there is a drug prescription, the drugs are delivered to the patient’s home by express delivery or can be obtained by the patient in the hospital. If a laboratory or imaging examination is needed, patients go to the hospital for the tests within the time determined by the responsible personnel of the internet hospital via a text message. If hospitalization is required, patients can come to the hospital for the admission procedure at the time determined by the responsible personnel of the internet hospital via a text message (Figure 2).

**Figure 3:**
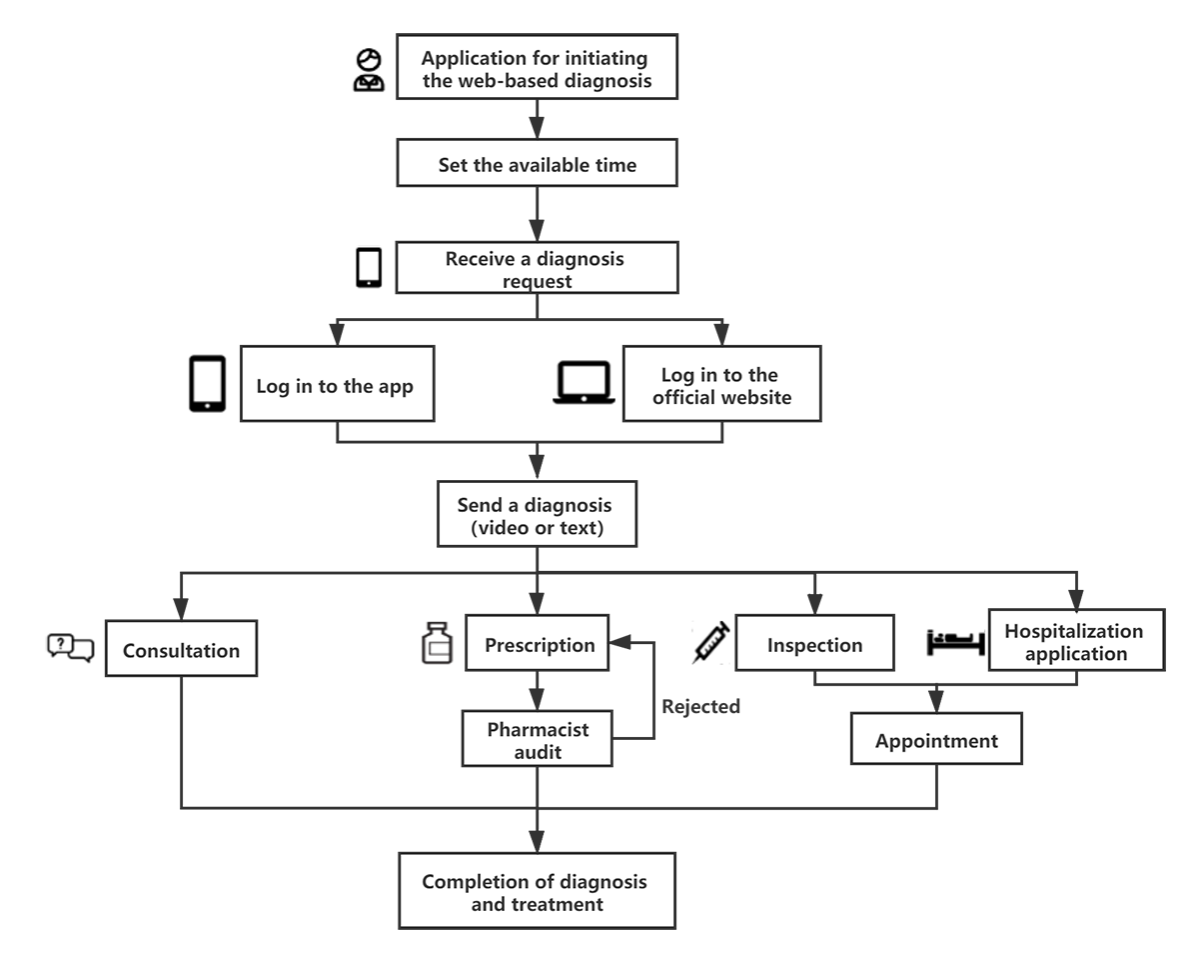
Flowchart of web-based clinic medical services for doctors.

Doctors can set their available times for the web-based clinic in their spare time upon obtaining permission to conduct web-based clinics. Doctors access the web-based diagnosis clinic in the physician version of the “Online FAHZU” app or log in to the official website of The First Affiliated Hospital, Zhejiang University to access the web-based clinic. After checking the patient’s basic information and medical records, the doctor launches a video call with the patient. After the diagnosis, the doctor completes the diagnosis and writes down notes in the medical record. The doctor can also issue online applications for drugs, inspections, and hospitalizations. The diagnosis can be completed after the doctor previews the medical advice. If the prescription is not approved by the pharmacist, it can be revised for approval (Figure 3).

### Online Appointments

Unlike other web-based communication platforms that merely provide consultation services, patients of FAHZU Internet Hospital can come to the offline hospital for inspection with an inspection sheet opened by the internet hospital at the inspection time provided via text message by the responsible internet hospital personnel. If the patient is identified for hospitalization while visiting the internet hospital, the physician issues a hospitalization certificate. The patient is admitted to the hospitalization appointment process to await notification from the hospital.

### Patient Follow-Up

A discharge follow-up center is subordinate to the internet hospital. The responsible nurse of the ward calls the discharged patient for follow-up inquiry. Any patients who require subsequent visits and inspection appointments are connected to the internet hospital and reexamined by a corresponding physician.

### Telemedicine

The internet hospital has remote cooperation with 206 county-level hospitals, 322 community service centers, and 64 pharmacies in China to provide teleconsultation and referral services.

### Routine Operation and Management

Full-time staff in the Internet Hospital Office are responsible for the management and routine operation of the internet hospital led by the director of the hospital’s medical department. After receiving doctors’ applications to initiate a web-based clinic, the Internet Hospital Office takes charge of initiating the clinic for candidates after they are trained and qualified in maintaining personal information, authority management, and evaluation performance for doctors engaging in the web-based clinic. In addition to maintaining and ensuring the normal operation of the internet hospital’s web-based clinic, the platform status, patients’ registered information, diagnosis, drug prescription auditing status, distribution information, timely appointment check, and notification to patients via text message, physician-patient phone consultations are monitored through the backstage operation. The information department, pharmacy department, various inspection departments, medical insurance office, outpatient office, and departments that are closely related to the operation of the internet hospital should be well coordinated with staff cooperation. The hospital staff should also work together with the quality management office of the hospital to formulate related management systems, service processes, emergency plans, and job responsibilities by referring to administration regulations and specifications issued by the government for the internet hospital. All text messages, informed consent forms, FAQs, and similar files should be prepared in accordance with work requirements. All of these processes should be constantly optimized as per actual situations at work. After inquiring about patients’ and doctors’ use experience of the internet hospital, related departments should address the items to be improved in a timely manner to enhance the diagnosis and treatment processes by continuously optimizing the system and related functions.

### Data Management

**Figure 4:**
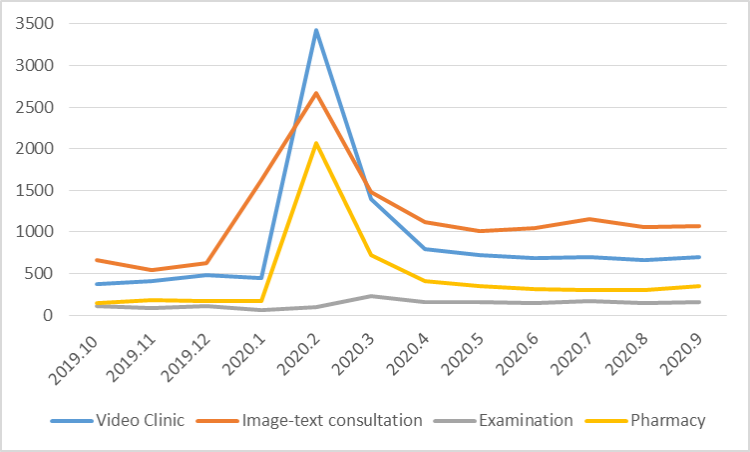
Number of patients in internet hospitals in the past year.

Statistics are compiled monthly on online outpatient visits, including basic information of patients, department distribution, visiting purposes, drug prescription and distribution ratio, prescription inspection and the completion ratio of inspections, and patent satisfaction. Based on these data, the internet hospital can be continuously improved. Figure 4 shows the number of patients that received services from the internet hospital from October 2019 to September 2020. From January to April 2020, due to the COVID-19 epidemic, the number of patients using the internet hospital soared. Since then, the epidemic in China has been stable, and most patients have returned to offline treatment; however, the overall number has increased (Figure 4).

### Publicity and Promotion

The internet hospital is promoted in a variety of ways. For example, an internet hospital experience center is established in the offline hospital clinic brochures about the internet hospital and doctors’ business cards are distributed by staff, plastic bags designed to promote the internet hospital are placed in the pharmacy, and holders for medical records with slogans are put in the outpatient reception area.

### Visit Reception and Experience Sharing

Since its foundation, FAHZU Internet Hospital has shared its working methods and operational experiences with more than 1000 medical institutions worldwide. The FAHZU Internet Hospital team also provided reference opinions for the Administration Regulations on Internet Hospitals issued by the Chinese government in 2018.

### Ensuring Network Security

**Figure 5:**
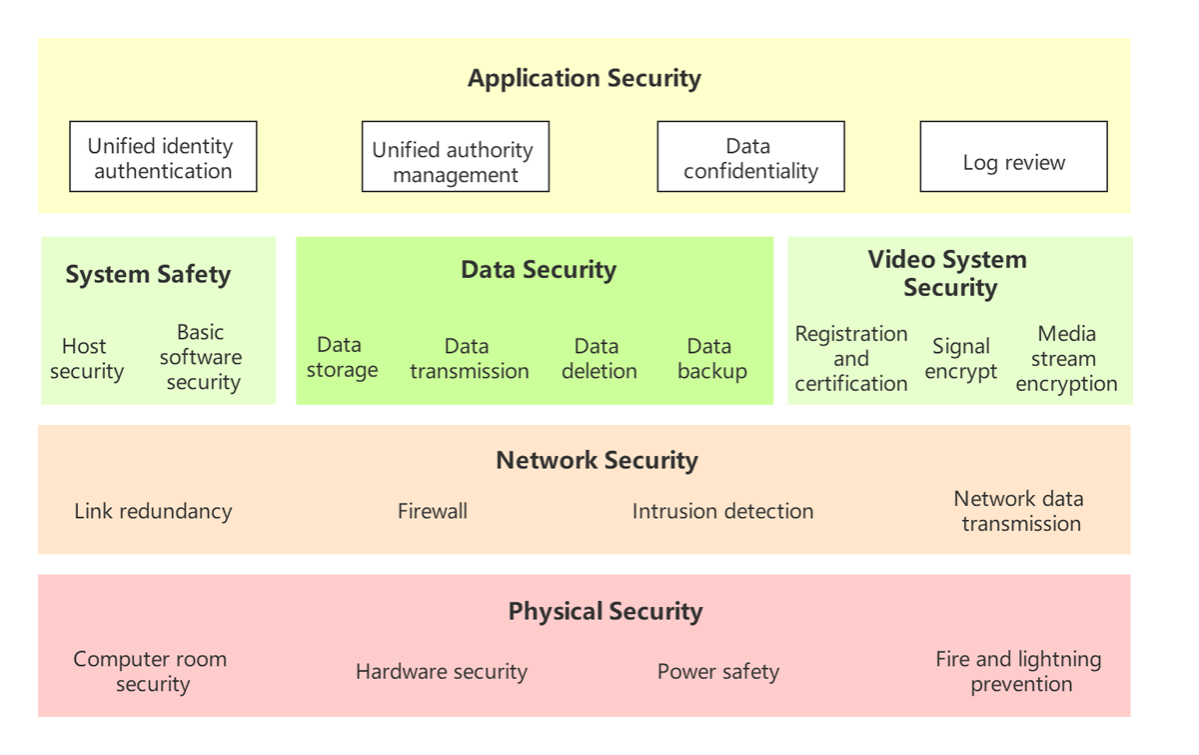
Safety management and standards.

In accordance with the “Guidance on Classified Protection of Information Security in the Health Industry,” “Basic Requirements for Hierarchical Protection of Information Security Technology Information Systems,” and “Technical Solutions for the Construction of Hospital Information Platforms Based on Electronic Medical Records” of the National Health Commission of China, we have made efforts to develop a technical system that meets the requirements for physical security, network security, host security, app security, and data security. The management system is constructed according to five basic technical requirements: a security management system, security management organization, personnel safety management, system construction management, and system operation and maintenance management (Figure 5).

### Overview and Prospects

Since medical services and resources are clustered in large cities, it is difficult for patients in remote areas to access medical resources in large hospitals due to the limitations of economics, distance, and time [20-22]. In addition, it is inconvenient for patients with chronic diseases who live in cities to frequently visit offline hospitals for inspection and drugs (on a regular basis of 15 days or 1 month in China and every 3 months in some cases). Developing internet hospitals might be a good choice to address this dilemma. Patients can obtain quick access to better medical resources through web-based diagnosis and treatment without wasting transportation expenses and time waiting. In a health emergency such as the COVID-19 pandemic, internet hospitals can soothe people’s panic, screen out suspected patients, and avoid unnecessary gatherings of patients, which is a good way to supplement offline medical treatment [23]. A summary analysis of the effectiveness of China’s internet hospitals found that internet hospitals can ease the difficulty of seeking medical care for patients in remote areas, facilitate patients’ communication with doctors in a timely manner, and enable refined tracking of chronic diseases and postoperative patients, thereby improving the effectiveness of patients seeking medical care. At the same time, the convenient medical service experience leads to high patient satisfaction [24,25].

Of course, internet hospitals also have some limitations. First, medical treatment requires practical operations. The network cannot perform physical examinations on patients. Laboratory and imaging examinations also need to be completed offline. Incomplete online medical procedures may affect the accuracy of doctors’ judgments. Second, it is difficult for elderly patients who are not experienced with electronic equipment to see a doctor in this way. Third, limited by the hospital’s own influence and the influence of the majority of people who prefer the traditional mode, the number of patients in internet hospitals operated by offline hospitals is not growing rapidly.

FAHZU Internet Hospital is independently operated by a large hospital. Doctors, nurses, and management staff, who are the same as those in offline hospitals, can complete tasks that are difficult to complete on third-party platforms, including appointments for examinations and hospitalizations, making the patient treatment process smoother and more complete while controlling patient privacy and data security. However, we also identified some problems in our operations. First, although the patients who visited the clinic provided personal information, including medical insurance information, we were unable to verify whether they actually held this insurance. We should consider adding authentication methods such as face recognition and optical character recognition identification. Second, there is no standard consultation network process, which makes it difficult to guarantee the homogeneity of the work. Third, the relevant laws are not perfect, and there is no legal basis for many issues. Finally, although we train the doctors on how to protect the privacy of patients before they start their work, and the hospital has built an isolated internet hospital outpatient studio for doctors to provide online service during working hours, we cannot guarantee whether a doctor adequately protects the privacy of patients when providing online services outside of work.

Currently, internet hospitals are experiencing rapid growth in China owing to the strong support of the government. Since the First Affiliated Hospital, Zhejiang University has formed a mature operation and management system for FAHZU Internet Hospital that is subordinate to a large offline hospital with a long operating history, it is expected that our experience can provide a theoretical reference for other areas with interest in developing internet hospitals.

## Abbreviations

FAHZU: First Affiliated Hospital of Zhejiang University School of Medicine
